# Miller Fisher Syndrome in Morocco: A 14-Year Retrospective Study of 27 Cases at a Tertiary Neurology Center

**DOI:** 10.7759/cureus.107115

**Published:** 2026-04-15

**Authors:** Soumia Ait Ami, Yasmina Zakaria, Khaoula Balili, Mohamed Chraa, Nissrine Louhab

**Affiliations:** 1 Neurology, Centre Hospitalo Universitaire Mohammed VI, Marrakech, MAR; 2 Faculty of Medicine and Pharmacy, Cadi Ayyad University, Marrakech, MAR

**Keywords:** anti-gq1b antibodies, ataxia, guillain-barré syndrome, miller fisher syndrome (mfs), morocco, ophthalmoplegia

## Abstract

Introduction: Miller Fisher syndrome (MFS) is a rare, acute immune-mediated neuropathy characterized by the classic triad of ophthalmoplegia, ataxia, and areflexia. Epidemiological and clinical data from North Africa remain scarce. The primary objective of this study was to provide a comprehensive description of the epidemiological, clinical, neurophysiological, and outcome characteristics of MFS in a Moroccan population. The secondary objective was to analyze these findings in comparison with existing literature.

Methods: We conducted a retrospective descriptive study in the Neurology Department of Mohammed VI University Hospital in Marrakech, Morocco, over a 14-year period (January 2010-December 2024). Patients were included based on the presence of the classical clinical triad or compatible clinical features supported by cerebrospinal fluid (CSF) findings and anti-GQ1b antibody testing.

Results: A total of 27 patients were included (mean age 40.2 ± 16.1 years, range 7-70). A female predominance was observed (n=17, 63%; male-to-female ratio: 0.59), and a seasonal peak occurred during winter in 14 patients (50%). Antecedent infections were reported in 15 patients (55.6%), most commonly respiratory infections (33%). The complete clinical triad was present in eight patients (29.6%). Areflexia was most frequent (77.7%), followed by ataxia (70.3%) and ophthalmoplegia (66.7%). Motor weakness and sensory disturbances were observed in 51.9% and 55.6% of patients, respectively. Albuminocytologic dissociation was found in 59.3% of cases, and anti-GQ1b antibodies were positive in 69.2% of patients, as all patients underwent antibody testing. Regarding treatment, 59.3% received intravenous immunoglobulins and 25.9% underwent plasmapheresis; outcomes were favorable, with complete recovery in all patients at six months.

Conclusion: This study represents one of the largest reported series of MFS from North Africa and provides a detailed descriptive characterization of the disease in a Moroccan cohort. While trends such as female predominance and winter peak were observed, these should be interpreted with caution due to the small sample size and retrospective design. Similarly, although most patients received immunotherapy and achieved full recovery, causal inferences regarding treatment efficacy cannot be drawn. Despite frequent overlap with Guillain-Barré syndrome manifestations, the overall prognosis in this cohort was excellent.

## Introduction

Miller Fisher syndrome (MFS), first described by Charles Miller Fisher in 1956, is a distinct clinical variant of Guillain-Barré syndrome (GBS). It is an acute immune-mediated polyradiculoneuropathy characterized by the classical triad of ophthalmoplegia, ataxia, and areflexia [1].

The pathophysiology of MFS is thought to involve molecular mimicry triggered by antecedent infections. Several infectious agents, most notably *Campylobacter jejuni*, can induce the production of antiganglioside antibodies, particularly anti-GQ1b antibodies. These antibodies target gangliosides highly expressed in the paranodal regions of oculomotor nerves and muscle spindle afferents, thereby explaining the characteristic clinical manifestations of the syndrome [2].

MFS is considered a rare neurological disorder, with an estimated annual incidence of approximately 0.09 cases per 100,000 individuals. However, its epidemiological distribution shows significant geographic variability [3,4]. Large cohorts have been reported mainly in East Asia, particularly in Japan and Taiwan, as well as in Europe. In contrast, epidemiological and clinical data from North Africa remain limited, restricting the understanding of potential regional differences in clinical presentation and immunological profiles [4].

The primary objective of this study was to provide a comprehensive description of the epidemiological, clinical, electrophysiological, and outcome characteristics of patients diagnosed with MFS in a Moroccan tertiary neurology center over a 14-year period. The secondary objective was to analyze these findings in relation to existing literature, in order to highlight specific features of our cohort and contribute to a better understanding of the variability of this rare GBS variant.

## Materials and methods

This was a retrospective descriptive study conducted in the Neurology Department of Mohammed VI University Hospital in Marrakech, a tertiary referral center serving the Marrakech-Safi region of Morocco. The study period extended over 14 years, from January 2010 to December 2024.

The study was approved by the Mohammed VI University Hospital, Marrakech. As this was a retrospective study based on medical record review, the requirement for informed consent was waived. The study was conducted in accordance with the Declaration of Helsinki.

Study population

Medical records of all patients admitted with a diagnosis of MFS were retrospectively reviewed. Patients were included if they presented with the classical form of MFS, defined by the presence of the typical clinical triad of ophthalmoplegia, ataxia, and areflexia, or with incomplete forms such as acute ataxic neuropathy without ophthalmoplegia or acute ophthalmoparesis without ataxia, provided that these presentations were supported by biological and/or electrophysiological findings. Patients with overlap syndromes, defined as MFS associated with features of classical GBS (e.g., limb weakness or bulbar involvement), were also included.

The diagnosis was supported by cerebrospinal fluid (CSF) analysis demonstrating albuminocytologic dissociation and/or the presence of serum anti-GQ1b antibodies. These diagnostic criteria were consistent with established clinical definitions reported in the literature. All included patients met a minimum diagnostic threshold based on a compatible clinical presentation supported by CSF findings and/or anti-GQ1b antibody positivity.

Patients with incomplete medical records or alternative diagnoses explaining the neurological presentation, such as brainstem stroke or Wernicke’s encephalopathy, were excluded.

Data collection

Data were retrospectively collected from medical records using a predefined standardized data extraction form developed for this study to ensure consistency. This structured form was used to systematically collect the following variables: (i) Epidemiological data: age, sex, geographical origin, and season of onset; (ii) Clinical data: antecedent infections (respiratory or gastrointestinal) and recent vaccinations within four weeks prior to symptom onset, interval between symptom onset and hospital admission, and detailed neurological examination findings (cranial nerve involvement, motor and sensory deficits, deep tendon reflexes, and ataxia); (iii) Paraclinical data: electroneuromyography (ENMG), cerebrospinal fluid analysis (protein level and cell count), anti-ganglioside antibody testing, and neuroimaging studies (MRI or CT scan) when available; (iv) Therapeutic and outcome data: type of immunotherapy (intravenous immunoglobulins or plasmapheresis), duration of hospitalization, and clinical outcomes assessed at one and six months.

Laboratory analysis

Serum anti-GQ1b antibodies were detected using enzyme-linked immunosorbent assay (ELISA) techniques, performed according to the protocols available at our institution during the study period. All patients included in the study underwent anti-GQ1b antibody testing.

Electrophysiological studies

Electroneuromyography (ENMG) was performed using standard neurophysiological techniques. Motor and sensory nerve conduction studies were conducted, assessing parameters including conduction velocities, amplitudes, and distal latencies. The findings were interpreted according to conventional electrophysiological criteria to identify features consistent with demyelinating or axonal neuropathies. ENMG results were classified according to the predominant electrophysiological pattern (normal, demyelinating, axonal, or mixed).

Statistical analysis

Statistical analyses were performed using IBM SPSS Statistics for Windows, version 27.0.1 (IBM Corp., Armonk, New York, United States) and Microsoft Excel (Microsoft Corporation, Redmond, Washington, United States). Quantitative variables were expressed as means ± standard deviation (SD) and ranges, while qualitative variables were presented as frequencies and percentages. Given the descriptive nature of the study and the limited sample size, no inferential statistical analyses or subgroup analyses were performed.

## Results

Epidemiological characteristics

**Figure 1:**
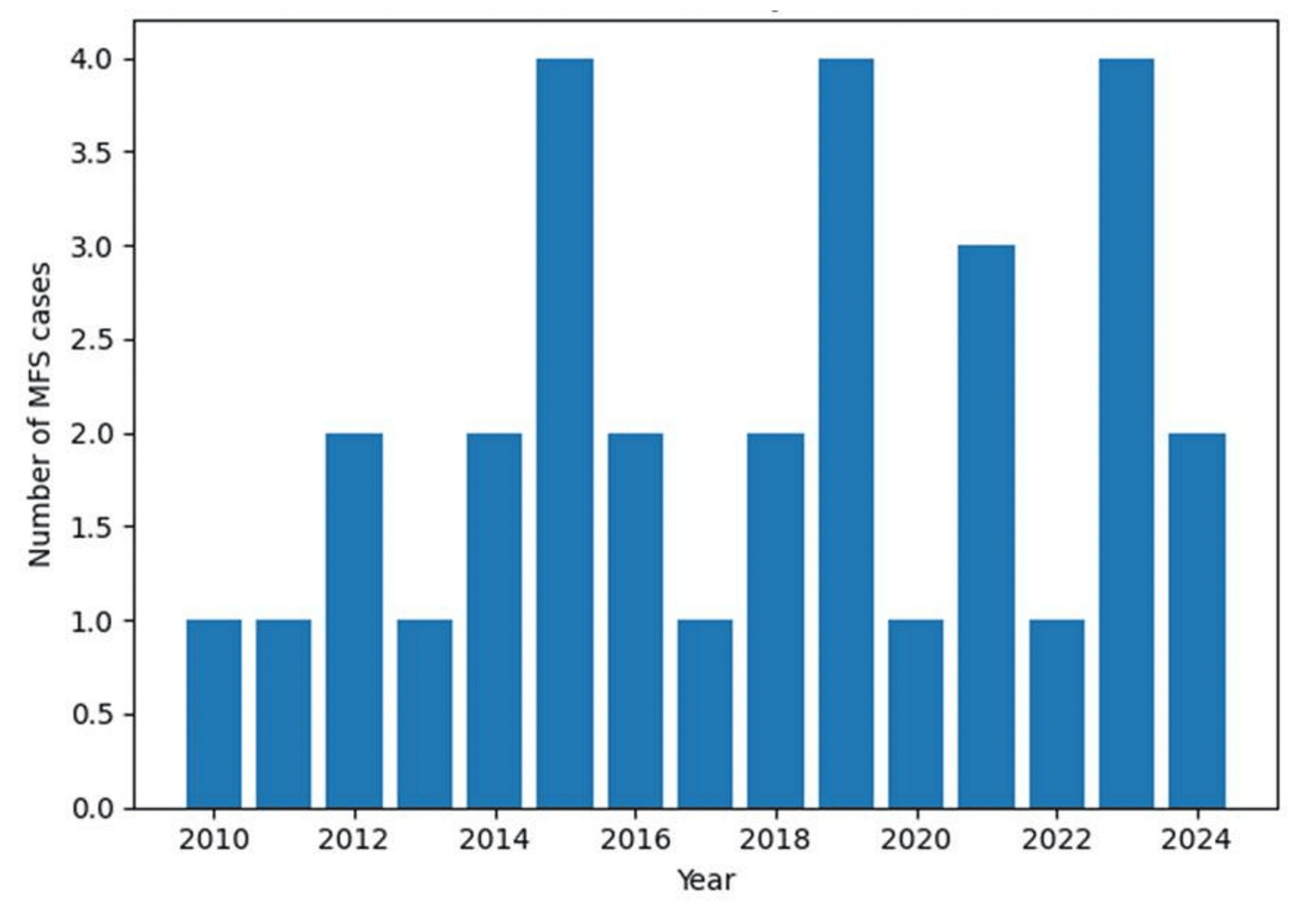
Annual distribution of Miller Fisher syndrome (MFS) cases from 2010 to 2024 (N = 27) Annual distribution of Miller Fisher syndrome cases (2010-2024), showing peaks in 2016, 2020, and 2024.

During the study period, a total of 27 patients were diagnosed with MFS, corresponding to an average of 1.8 cases per year. Peaks in incidence were observed in 2016, 2020, and 2024, with four cases recorded in each of these years (Figure 1).

The mean age of the patients was 40.2 ± 16.1 years (range: 7-70 years), with the majority of patients belonging to the age group of 30-59 years. A female predominance was observed, with 17 female patients (63%) compared to 10 male patients (37%), resulting in a male-to-female ratio of 0.59.

A marked seasonal distribution was identified, with 14 cases (50%) occurring during winter, followed by eight cases (29.6%) in spring and five cases (18.5%) in summer. No cases were recorded during autumn; however, this finding may reflect the limited sample size rather than a true seasonal absence. The highest monthly frequency was observed in March, accounting for six cases (22.2%).

A preceding infectious episode within four weeks before neurological onset was reported in 15 patients (55.6%). These infections were mainly respiratory tract infections in nine patients (33.3%) and gastrointestinal infections in five patients (18.5%). Two patients (7.4%) reported a recent COVID-19 vaccination.

Clinical presentation

**Figure 2:**
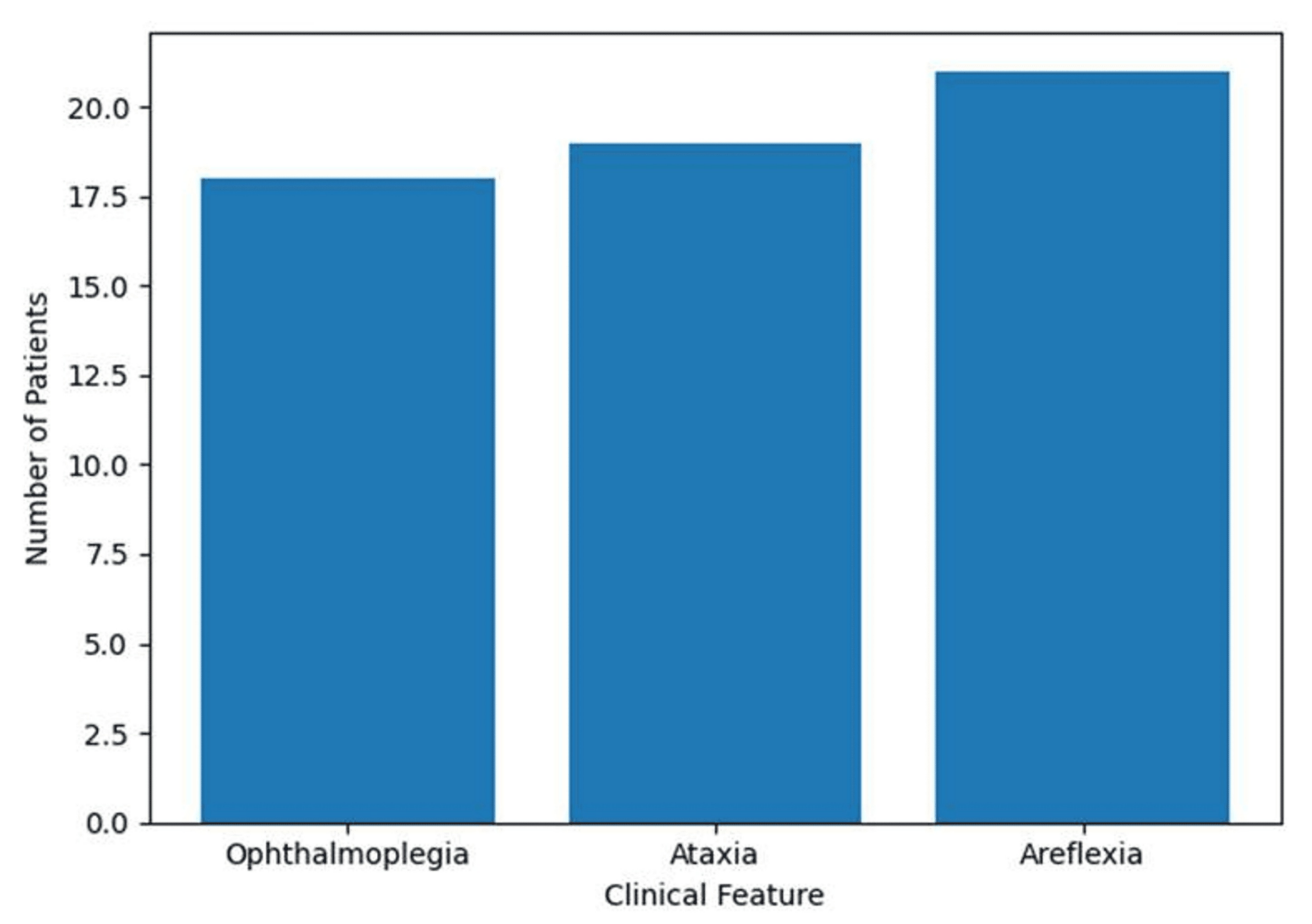
Distribution of Miller Fisher clinical triad

The mean interval between symptom onset and hospital admission was 10.4 ± 7 days. The complete classical triad of MFS (ataxia, areflexia, and ophthalmoplegia) was present in eight patients (29.6%) (Figure 2).

Based on clinical presentation, patients were categorized into classical, incomplete, and overlap forms. Incomplete forms of MFS were observed in 15 patients (55.6%), including acute ataxic neuropathy and isolated ophthalmoparesis. Overlap syndromes with GBS features were identified in four patients (14.8%), characterized by the presence of limb weakness and/or bulbar involvement. All included patients met a minimum diagnostic threshold based on compatible clinical features supported by cerebrospinal fluid findings and/or anti-GQ1b antibody positivity.

Ophthalmoplegia was observed in 18 patients (66.7%). It was bilateral in 17 patients (94.4%) and predominantly consisted of incomplete external ophthalmoplegia in 16 patients (88.9%). The most frequently affected cranial nerve was the abducens nerve (VI), involved in 15 patients (55.6%), followed by the oculomotor (III) and facial (VII) nerves, each affected in 14 patients (51.9%).

Ataxia was present in 19 patients (70.3%), resulting in significant gait instability. Areflexia was the most frequent clinical sign, observed in 21 patients (77.7%), while hyporeflexia was noted in five patients (18.5%). Motor weakness was present in 14 patients (51.9%). The deficit was symmetrical in all cases and predominantly involved the lower limbs. Tetraparesis occurred in six patients (22.2%), while tetraplegia was observed in four patients (14.8%).

Sensory disturbances were reported in 15 patients (55.6%), mainly presenting as paresthesia or hypoesthesia in a glove-and-stocking distribution (Table 1).

**Table 1:** Frequency of clinical manifestations in the study population CN: cranial nerve

Clinical Signh	Frequency (Percentage)
Areflexia	21 (77.7%)
Ataxia	19 (70.3%)
Ophthalmoplegia	18 (66.7%)
Sensory disturbances	15 (55.6%)
Motor deficit	14 (51.9%)
CN VI palsy	15 (55.6%)
CN III / VII palsy	14 (51.9%)

Paraclinical Investigations

Paraclinical investigations included electrophysiological studies, CSF analysis, and anti-GQ1b antibody testing. ENMG was performed in all patients. The electrophysiological findings were classified according to the predominant pattern. A demyelinating pattern compatible with acute inflammatory demyelinating polyneuropathy was observed in 13 patients (48.1%). A mixed axonal-demyelinating pattern was identified in four patients (14.8%), while five patients (18.5%) had normal findings. An axonal pattern was observed in one patient (3.7%). F-wave responses were normal in 20 patients (76.9%), whereas absent or prolonged F-waves were observed in the remaining cases, suggesting proximal nerve involvement. Detailed nerve conduction study findings from a representative patient are presented in Table 2.

**Table 2:** Nerve conduction study (NCS) results in a representative patient NCS demonstrated features consistent with a demyelinating polyradiculoneuropathy. Motor studies revealed prolonged distal latencies and reduced conduction velocities, particularly in the tibial nerves (37–39 m/s). In contrast, sensory nerve conduction studies were relatively preserved, with normal amplitudes and velocities. These findings support the diagnosis of an acute inflammatory demyelinating polyradiculoneuropathy within the spectrum of Miller Fisher syndrome. Normal conduction velocity is ≥50 m/s in the lower limbs and ≥55 m/s in the upper limbs.

Nerve	Stimulation Site	Latency (ms)	Amplitude (Motor: mV/ Sensory: µV)	Conduction Velocity (m/s)	Normal Amplitude (mV)
Peroneal motor – right	Ankle	4.3	3.7 mV	45	2–6 mV
Peroneal motor – right	Below the Knee	13.2	3.1 mV	-	2–6 mV
Peroneal motor – left	Ankle	5.3	4.0 mV	42	2–6 mV
Peroneal motor – left	Below the Knee	12.9	3.3 mV	-	2–6 mV
Tibial motor – right	popliteal segment	9.7	2.7 mV	37	4–20 mV
Tibial motor – left	popliteal segment	9.2	2.3 mV	39	4–20 mV
Median motor -right	Wrist	9.6	3.3 mV	51	4–15 mV
Median motor -right	Elbow	13.4	2.5 mV	-	4–15 mV
Median motor – left	Wrist	6.6	4.2 mV	40	4–15 mV
Median motor – left	Elbow	12.2	2.9 mV	-	4–15 mV
Ulnar motor – right	Wrist	5.6	10.9 mV	47	6–12 mV
Ulnar motor – right	Below the elbow	10.3	10.5 mV	-	6–12 mV
Ulnar motor – right	Above the elbow	7.2	10.1 mV	-	6–12 mV
Ulnar motor – left	Wrist	6.6	10.0 mV	45	6–12 mV
Ulnar motor – left	Below the elbow	10.6	10.2 mV	-	6–12 mV
Ulnar motor – left	Above the elbow	7.7	10.1 mV	-	6–12 mV
Median sensory –right	Wrist	2.4	41.3 µV	50	≥20 µV (sensory)
Median sensory – left	Wrist	2.7	48.0 µV	46	≥20 µV (sensory)
Ulnar sensory – right	Wrist	2.1	16.7 µV	48	≥17 µV (sensory)
Ulnar sensory – left	Wrist	2.4	19.9 µV	49	≥17 µV (sensory)
Sural sensory – right	Ankle	5.0	9.6 µV	48	≥6 µV (sensory)
Sural sensory – left	Ankle	4.7	8.4 µV	50	≥6 µV (sensory)

Lumbar puncture was performed in all patients. Albuminocytologic dissociation was observed in 16 patients (59.3%). Elevated CSF protein levels (>0.45 g/L) were detected in 16 patients (61.5%), with a mean protein concentration of 0.87 g/L (range: 0.26-3.49 g/L). CSF cell counts remained within normal limits in 25 patients (92.6%). Anti-GQ1b antibodies were positive in 69.2% of patients, as all patients underwent antibody testing.

Management and prognosis

The mean delay between symptom onset and initiation of treatment was 14.4 ± 7 days. Intravenous immunoglobulins were administered in 16 patients (59.3%) at a dose of 0.4 g/kg/day for five days. Plasma exchange was performed in seven patients (25.9%), while sequential therapy combining intravenous immunoglobulins and plasma exchange was used in one patient (3.7%). Supportive treatment alone was sufficient in three patients (11.1%) due to spontaneous clinical improvement.

The clinical course was favorable in all patients. The mean time to the onset of neurological improvement was 11.3 ± 7.7 days. At one month of follow-up, 24 patients (88.9%) had achieved complete recovery, while minor residual deficits persisted in three patients (11.1%). At six months, complete functional recovery was observed in all patients (27 patients, 100%). No relapses or deaths were recorded during the follow-up period.

## Discussion

Epidemiological characteristics

Our study highlights several distinctive epidemiological features of MFS in the Moroccan population. The mean age at onset in our cohort (40.2 years) is consistent with that reported in international studies, where the average age typically ranges between the fourth and fifth decades of life [1,2].

Interestingly, we observed a clear female predominance, whereas most studies report a slight male predominance [3]. Recent analyses of anti-GQ1b antibody syndrome cohorts suggest a more balanced sex distribution, indicating that regional variations may exist depending on genetic or environmental factors [4]. A seasonal variation was also observed, with a marked winter predominance. Antecedent infections were frequently reported, particularly respiratory infections, supporting the role of infectious triggers in the pathogenesis of MFS. This finding is consistent with recent literature highlighting the involvement of viral and bacterial infections in initiating autoimmune responses through molecular mimicry [5].

Clinical spectrum

The complete classical triad of ophthalmoplegia, ataxia, and areflexia was observed in only eight patients (29.6%). Although lower than in some series, this finding likely reflects the inclusion of incomplete forms and overlap syndromes in our cohort. Increasing evidence suggests that MFS belongs to a broader spectrum of anti-GQ1b antibody-mediated disorders, including GBS variants and Bickerstaff brainstem encephalitis [6].

In our study, limb weakness was observed in 14 patients (51.9%), indicating a relatively high rate of overlap with GBS. Similar findings have been reported in recent studies analyzing large cohorts of patients with anti-GQ1b antibody syndromes, which demonstrate considerable clinical heterogeneity and overlapping phenotypes [4].

Ophthalmoplegia was observed in 18 patients (66.7%), which is slightly lower than the prevalence reported in several international series. One possible explanation is the delay between symptom onset and hospital consultation in our setting. Since ophthalmoplegia often represents one of the earliest symptoms to improve, partial recovery may have occurred before admission.

Diagnostic biomarkers

Anti-GQ1b antibodies represent the immunological hallmark of MFS and play a central role in its pathogenesis. Recent studies confirm that these antibodies are present in a large proportion of patients and are strongly associated with ophthalmoplegia and other cranial nerve manifestations [7]. However, the prevalence of anti-GQ1b antibodies may vary depending on geographic region and testing methods. A recent study conducted in China reported variable positivity rates and highlighted possible epidemiological differences related to regional factors or the COVID-19 pandemic [8].

Electrophysiological findings in MFS are heterogeneous. While demyelinating patterns are common, normal nerve conduction studies may also be observed, particularly in the early stages of the disease. This variability has been widely reported in recent neurophysiological studies of anti-GQ1b antibody syndromes [4].

Therapeutic considerations and prognosis

There is currently no consensus regarding the systematic use of immunotherapy in mild forms of MFS, as the disease is generally self-limited and associated with a favorable prognosis. Intravenous immunoglobulin and plasma exchange are commonly used in clinical practice, particularly in patients with severe symptoms or overlap with GBS [6,9].

In our cohort, three patients (11.1%) recovered completely with supportive management alone, suggesting that immunotherapy may accelerate neurological recovery but does not necessarily alter long-term outcomes. Previous studies have also reported excellent recovery rates, with most patients achieving full functional recovery within several months [4,10].

Strengths and limitations

This study presents several notable strengths. Its long duration of 14 years and the comprehensive clinical and paraclinical characterization of patients provide a detailed picture of MFS in a North African population, a region for which data are currently scarce. The inclusion of real-world cases enhances the clinical relevance of our findings, and the outcome data are clearly documented, contributing valuable descriptive information to the literature.

However, several limitations must be acknowledged. The study is limited by its small sample size and retrospective single-center design, which restricts the generalizability of our findings. Heterogeneity in clinical presentation, including incomplete and overlap forms, introduces variability in interpretation. The lack of standardized diagnostic and methodological protocols over the 14-year period may affect reproducibility, and potential selection and information bias inherent to retrospective record review could influence data completeness and accuracy. Finally, the analysis was primarily descriptive, limiting the exploration of associations, predictors, or statistical comparisons.

Despite these limitations, the study provides valuable descriptive insights into the epidemiology, clinical features, electrophysiology, and outcomes of MFS in Morocco. We have interpreted the findings with caution, particularly regarding apparent epidemiological differences, to avoid overgeneralization given the cohort size. Overall, this study contributes meaningfully to the understanding of MFS in an underrepresented population while highlighting areas for future research.

## Conclusions

MFS remains a rare but well-defined variant of GBS. Our 14-year retrospective study highlights the clinical spectrum, electrophysiological findings, and generally favorable outcomes of MFS in a Moroccan tertiary neurology center. Incomplete forms are relatively frequent and may pose diagnostic challenges. While descriptive trends such as female predominance and a winter peak were observed, these findings should be interpreted with caution due to the small sample size and retrospective single-center design. Similarly, although most patients received immunotherapy and achieved complete recovery, the study design does not allow causal inferences regarding treatment efficacy. Early recognition of MFS remains essential to avoid unnecessary investigations and ensure appropriate management. Further multicenter studies are warranted to better characterize the epidemiological and immunological features of MFS in North Africa.
